# Serum periostin level is not sufficient to serve as a clinically applicable biomarker of osteoarthritis

**DOI:** 10.1186/s12891-022-06017-x

**Published:** 2022-12-01

**Authors:** Qizhao Tan, Zhongwei Yang, Xing Xin, Bin Yang, Zhili Xing, Feng Li, Ke Zhang, Yun Tian, Tengjiao Zhu

**Affiliations:** 1 Department of Orthopaedics, Peking University Third Hospital, Haidian District, 49 North Garden Road, Beijing, 100191 P.R. China; 2 Department of Orthopaedics, Zibo Central Hospital, Zibo, Shandong, 255000 P.R. China; 3 Engineering Research Center of Bone and Joint Precision Medicine, Ministry of Education, Beijing, P.R. China; 4 Department of Orthopaedics, Peking University International Hospital, Beijing, 102206 P.R. China

**Keywords:** Osteoarthritis, Biomarkers, Knee joint, Radiography, Risk factors

## Abstract

**Background:**

Emerging knowledge has highlighted the role of periostin (POSTN) in osteoarthritis (OA) process; however, whether POSTN is suitable as a biomarker of OA remains unclear. This study aimed to investigate the potential value of POSTN as a biomarker of OA.

**Methods:**

Ten 6-month-old female Sprague–Dawley (SD) rats were used in this study. Five rats underwent ovariectomy (OVX) operation and the others were carried out sham operation. Thirty-two patients with OA and eighteen patients who had meniscus injuries or ligament injuries but with intact articular cartilages were recruited in this study from January to July 2019 at the Peking University International Hospital. We first detected the expression of POSTN in the cartilage of OVX induced OA rats and different compartments of the knee joint in patients with OA using immunohistochemistry. Besides, serum POSTN levels in patients with or without OA were examined using enzyme-linked immunosorbent assay (ELISA). The associations among serum POSTN levels, clinical symptoms, and radiological severity were assessed according to the Western Ontario and McMaster Universities Osteoarthritis Index (WOMAC) scores and, Kellgren-Lawrence (KL) grading, respectively. Finally, multivariable cumulative link models were established to evaluate the validity of serum POSTN level as a predictor of knee OA.

**Results:**

The significantly higher POSTN expression was found in OVX-OA rats than Sham rats, while, the expression of POSTN was significantly higher in the torn cartilage of patients with OA. However, the serum POSTN level did not differ significantly between patients with and without OA. Additionally, we found no remarkable associations between serum POSTN level and WOMAC scores and KL grading. Subsequent analysis revealed that serum POSTN was not a significant predictor of OA.

**Conclusion:**

Thus, although POSTN may be involved OA process and local POSTN is valuable in disease diagnosis and distinguishing of the severity of disease, its serum level is not sufficient to serve as a candidate biomarker of OA given the current analysis technology.

**Supplementary Information:**

The online version contains supplementary material available at 10.1186/s12891-022-06017-x.

## Background

Osteoarthritis (OA) is characterized by progressive degeneration of cartilage, accompanied by synovitis, subchondral bone remolding, and damage to other joint structures. It has a worldwide prevalence and is the third leading cause of disability in the elderly [1, 2]. However, the pathophysiology of OA remains unclear. OA was recognized as systemic and local low inflammatory states [3]. Moreover, the metabolic changes such as intestinal microbiota changes also are associated with the pathogenesis of OA [4].

The diagnosis of OA mainly relies on classically clinical symptoms and radiography, which usually reveals OA at the end-stage. Molecular changes have been reported to appear much earlier than the apparent clinical symptoms [5]. Hence, biomarkers of OA diseases has been a popular research trends [6]. Update, a number of biochemical markers of OA, such as C-telopeptide of cross-linked collagen type II, cartilage oligomeric matrix protein, matrix metalloproteinase-3, have been proposed to help measure both the progression of the disease and, potentially, assess the effectiveness of new treatments [7, 8]. However, to date, there have been no clinically applicable biomarkers in the diagnosis, monitoring, and prognosis of OA due to the heterogeneity in the pathogenesis and multiformity of the clinical symptoms [9].

Periostin (POSTN), belonging to the family of fasciclins, is a disulfide linked 90-kDa heparin-binding N terminus-glycosylated protein, which originates from the osteoblasts [10]. It was initially identified in the periosteum and bone [11]. The role of POSTN has been widely researched and the protein has known function in tissue repair [12], accelerating development of tumors [13] and bronchial asthma [14], and various inflammatory settings. In recent years, POSTN is found to be related to OA [15, 16]. It has been demonstrated that POSTN was upregulated in OA tissues [17]. A microarray analysis conducted by loeser et al. revealed high deposition of POSTN on the surface and in the denatured cartilage matrix in a surgically induced mouse OA model [18]. A global gene expression profiling study reported that POSTN mRNA was upregulated in subchondral bone in a surgical OA rat model [19]. In addition, POSTN has been demonstrated to be present in osteocytes, osteoblasts, and in lining cells in osteoarthritic subchondral bone of patients with OA at mRNA and protein levels [20]. These findings prompted us to propose the hypothesis that POSTN may be responsible for the pathogenesis of OA. Furthermore, a previous study reported that synovial fluid POSTN levels were positively correlated with the radiographic severity of knee OA [21]. Therefore, POSTN could be considered as an additional prognostic marker of certain stages and types of OA. However, the significance predictive value of circulating POSTN in the patients with OA have received little attention. Whether POSTN is a suitable biomarker of OA remains controversial.

Therefore, in the present study, we aimed to assess the rationality of POSTN as a potential biomarker for OA. First, POSTN involvement in OA process was investigated by detecting the expression level of POSTN in the cartilage of ovariectomy (OVX)-OA rats. Next, the involvement of POSTN in severity-related OA progression was explored by detecting the expression of POSTN in the damaged cartilage of the medial compartment and intact cartilage of the lateral compartment in patients with OA. The systematic level of POSTN was evaluated in patients with and without OA using Enzyme-linked immunosorbent assay (ELISA) to determine its suitability as a diagnostic marker. The clinical association between the serum POSTN level and symptomatic and radiographic severity of OA was also evaluated. Finally, multivariable cumulative link models were built to evaluate the validity of serum POSTN as a predictor of knee OA.

## Methods

### Animal preparation

Ten 6-month-old female Sprague–Dawley (SD) rats were used in this study. After 1 week of adaptive feeding, dual ovariectomy (*n* = 5) or Sham operation (*n* = 5) were performed. Ten weeks post-operation, all rats were sacrificed and knee joints were harvested for follow-up bone histomorphometric assessment and histological analysis.

To confirm the success of the OVX model, these samples were performed safranin O-fast green (Servicebio, CHN) stain according to the corresponding protocols [22]. The erosion severity scores of cartilage were used to evaluate cartilage surface erosion through which significant difference could be detected between Sham and OVX groups. The length of degraded cartilage and the total length of articular cartilage were measured by Image-Pro Plus (Version 6.0, Media Cybernetics, USA). The ratio of the length of the degraded cartilage surface to the total length of cartilage surface was calculated as erosion severity scores [23]. In addition, the microstructure of the subchondral bone of the tibia was analyzed using micro-CT. Briefly, all the knee joints were carefully excised and scanned by high-resolution Micro-CT (Inveon, Siemens Medical Solutions USA, Inc., IL, USA). The following parameters of the scan were set and a high-med resolution scan was carried out: the voltage at 80-kV voltage and the current at 500-μA current, and 360 projections per 360°. The scanned data were reconstructed using the Inveon Acquisition Workplace software. The tibia subchondral trabecular bone below the subchondral bone plate extending to the epiphyseal line was selected as a region of interest (ROI). The bone volume/total volume (BV/TV) of the ROI was calculated to evaluate the structure of the subchondral bone.

### Participant selection

Thirty-two patients with OA who underwent total keen joint arthroplasty (TKA) from January to July 2019 at the Peking University International Hospital orthopedics department were recruited. Patients with OA were diagnosed according to the American College of Rheumatology [24]. Eighteen patients in the arthroscopy surgery (ASS) group who had meniscus injuries or ligament injuries but with intact articular cartilages diagnosed by magnetic resonance imaging were selected as the control group. Detailed characterizations of the patients are summarized in Supplementary Table S1. Clinical data were reviewed to preclude any forms of secondary OA. The following patients were excluded: those with autoimmune diseases such as rheumatoid arthritis, systemic lupus erythematosus, and ankylosing spondylitis; those who had a history of knee injuries within 1 month of intra-articular drug injection 6 months before surgery; those who underwent TKA revision or simultaneous bilateral knee OA; those patients with OA in any other joints. The Ethics Committee at Peking University International Hospital approved the study protocol (authorization number: 2018–069[BMR]), and written informed consent was obtained from all patients before participation.

### Clinical cartilage sample collection

The tibial plateaus was harvested from 32 end-stage patients with OA who underwent TKA. We differentiated the damaged cartilage zones in the medial plateau from the zones that were macroscopically intact in the lateral plateau. The macroscopically intact cartilage was considered as the control group (NC), and the lesion cartilage in the lateral plateau was viewed as the experimental group (AC).

### Histological analysis of POSTN

For immunostaining, the rats were decalcified in EDTA for 5 weeks and clinical samples for 6 weeks. Then, the samples from 15 clinical patients and all rats were dehydrated in a tissue automatic dehydration machine (Leica, Germany) and embedded in paraffin. The samples then were cut at a thickness of 5-μm. Slices were deparaffinized before rehydration in grade alcohol. Thereafter, slices were quenched in 0.3% hydrogen peroxide in methanol. The rehydrated sections were subjected to antigen retrieval in gastric enzyme for 30 min followed by incubation with primary antibodies against the mouse. Then, the slides were incubated with POSTN antibodies (Cat.No.19899–1-AP) for 2 h at 37 °C. The antigen − antibody interaction was detected using Chromogen diaminobenzidine kit (ZSGB-BIO, Beijing, China). Images were captured using a digital slide scanner (Hamamatsu, Japan). Image-Pro Plus (Version 6.0, Media Cybernetics, USA) was used to semi-quantitatively analyze the relative expression of the proteins in sections.

### Blood collection and serum level of POSTN detection

To investigate if the systematic POSTN was different between patients with and without OA, blood was collected from the patients who underwent TKA (*n* = 32) and those with meniscal or ligament injuries (*n* = 18). The patients who underwent TKA were considered as the OA group and the latter was referred to as the Non-OA group. Blood was centrifuged at 4 °C, 1500 r/min for 15 min to obtain the serum. The serum was stored at -80 °C for the subsequent use. A Human ELISA Kit (Cat.No.AG-45B-0004-KI01, Adipogen, China) was used to measure the serum level of POSTN according to the manufacturer’s protocol. The dilution curve was y = 2054.5x-149.3 and *R*^2^ = 0.9953. The intensity of the color reaction was measured at 450 nm. The assay range of this kit was78 pg/ml-5000 pg/ml. Four samples of known concentration of human periostin were assayed in replicates 8 times to test precision within an assay and the mean CV was 7.12%. Additionally, four samples of known concentration of human periostin were assayed in 4 separate assays to test precision between assays and the mean CV was 7.62%.

### Clinical symptom assessments

To further demonstrate the potential value of POSTN as a biomarker of OA, the clinical associations between POSTN and symptomatic and radiographic OA were evaluated. The joint symptoms of all participants were assessed using Western Ontario and McMaster Universities Osteoarthritis Index (WOMAC). The WOMAC score was assessed using questionnaires, and the following subgroups were self-reported: knee joint pain, stiffness, and physical dysfunction.

### Knee joint radiographic assessments

A 15-degree flexion, standing, anteroposterior view image was performed in the symptomatic knees of each participant. The radiographic severity of OA was assessed according to the Kellgren-Lawrence (KL) grading system [25]. The KL grading system was grouped into five grades, as previously reported. All preoperative radiographs were assessed by a single trained orthopedic specialist blinded to the patient’s clinical and laboratory data. Participants with radiographic knee OA of KL grade ≥ 2 in at least one knee were defined as patients with OA.

### Proportional odds logistic regression models

To assess the predictive role of serum POSTN for OA, an odds logistic regression model was constructed. We also determined several other potential factors for OA. All risk factors included in the analysis of the predictors of OA were followed and divided into patient-related and laboratory-related risk factors. Age, sex, and body mass index were chosen as patient-related factors. The following laboratory-related risk factors were assessed: C-reactive protein, erythrocyte sedimentation rate, white blood cell count, neutrophil ratio, hemoglobin, platelet count, total protein, albumin, alanine aminotransferase, aspartate aminotransferase, creatinine, prothrombin time, international normalized ratios, activated partial thromboplastin time, fibrinogen, thrombin time, fibrin degradation product, D-dimer.

For analysis, the diagnosis was labeled as an ordinal categorical variable with levels of 0 and 1. Level-1 represented OA and level-0 was defined as non-OA. The descriptive summaries of each variable are shown in Table 1, and the numeric variables are presented as mean ± standard deviation. Two proportional odds logistic regression models were used to assess the extent of covariates in the diagnosis of knee OA. First, Model1 integrated all factors to analyze the prediction of knee OA. Model 2 selectively assessed laboratory-related factors as a predictor. The Akaike information criterion (AIC) was used to evaluate the different models. Variable importance plots were obtained by ranking each variable by the increase in AIC upon its removal [26]. AIC ≥ 2 represents a statistically better model [27]. Odds ratios (ORs) were reported as the ratio between the 75th and the 25th percentiles and significance was set at *p* < 0.05. The statistical analysis above was performed using R software (R version 3.6.3).

**Table 1:** Predictors of knee osteoarthritis for model 1 (patient- and laboratory-related factors) and model 2 (laboratory-related factors)

Variable	Model 1		Model 2	
	OR (95% CI)	*p*-value	OR (95%CI)	*p*-value
Patient-related risk factors				
Age	1.02 (1.01 – 1.03)	< 0.001**		
Sex				
Female	0.86 (0.60 – 1.25)	0.444		
Male	—	—		
BMI	1.00 (0.98 – 1.02)	0.808		
Laboratory-related risk factors				
POSTN	1.00 (1.00 – 1.00)	0.910	1.00 (1.00 – 1.00)	0.440
CRP (mg/L)	1.01 (0.99 – 1.02)	0.569	1.00 (0.98 – 1.02)	0.961
ESR (mm/H)	1.00 (0.98 – 1.01)	0.720	0.99 (0.97 – 1.01)	0.317
WBC	1.05 (0.95 – 1.15)	0.345	1.04 (0.90–1.19)	0.601
Neu (%)	0.99 (0.98 – 1.01)	0.442	0.99 (0.97 – 1.01)	0.395
Hb	1.00 (0.99 – 1.01)	0.991	1.00 (0.98 – 1.01)	0.763
PLT	1.00 (1.00 – 1.00)	0.808	1.00 (0.99 – 1.00)	0.218
TP	1.00 (0.96 – 1.05)	0.937	1.04 (0.98 – 1.11)	0.210
ALB (g/L)	0.99 (0.93 – 1.06)	0.807	0.93 (0.85 – 1.02)	0.151
ALT (U/L)	1.00 (0.98 – 1.02)	0.839	0.99 (0.96 – 1.01)	0.275
AST (U/L)	1.00 (0.98 – 1.03)	0.763	1.01 (0.97 – 1.05)	0.594
Cr (mmol/L)	1.00 (1.00 – 1.00)	0.555	1.00 (1.00 – 1.00)	0.633
PT (S)	1.04 (0.86 – 1.26)	0.677	1.05 (0.81 – 1.37)	0.717
INR	1.03 (0.96 – 1.10)	0.450	1.03 (0.93 – 1.14)	0.571
APTT (S)	1.03 (0.97 – 1.10)	0.289	1.03 (0.95 – 1.12)	0.504
FIB	1.00 (1.00 – 1.00)	0.531	1.00 (1.00 – 1.01)	0.088
TT	0.99 (0.98 – 1.00)	0.227	1.00 (0.98 – 1.01)	0.587
FDP	0.96 (0.82 – 1.12)	0.618	0.90 (0.72 – 1.13)	0.365
D-dimer	1.00 (1.00 – 1.00)	0.650	1.00 (1.00 – 1.00)	0.388

*BMI *body mass index,* CRP* C-reactive protein, *ESR* erythrocyte sedimentation rate, *WBC* white blood cell count, *Neu%* neutrophil ratio, *Hb* hemoglobin, *PLT* platelet count, *TP* total protein, *ALT* alanine aminotransferase, *AST* aspartate aminotransferase, *Cr* creatinine, *PT* prothrombin time, *PT-A* plasma thromboplastin antecedent, *INR* international normalized ratios, *APTT* activated partial thromboplastin time, *FIB* fibrinogen, TT thrombintime, *FDP* fibrin degradation product ** indicates the statistical difference

### Statistical analysis

The data were analyzed using GraphPad Prism 7.0 (GraphPad Software, Inc., La Jolla, CA, USA) and results are presented as mean ± standard error of the mean in immunohistochemistry and ELISA. The results of immunohistochemistry and ELISA were analyzed using a non-parametric Kruskal–Wallis test, and *p* values < 0.05 were considered statistically significant. Linear regression analyses were used to examine the associations between serum POSTN level (the independent variable) and WOMAC scores and KL grading. The regression analyses performed were based on the assumption that the dependent variable was a function of the independent variables. Thus, the estimated associations were directional effects rather than causally undefined relations.

## Results

### The characterization of clinical samples

Of the enrolled participants for serum analysis, 32 patients with OA and 18 healthy subjects that were relatively well-matched in terms of BMI were selected. However, as expected, these two groups showed significant differences in age, CRP, time since diagnosis, WOMAC score, and K-L grade (Supplementary Table 1).

### Success of the OVX-OA rat model and increased POSTN expression in OVX-OA rats

**Fig. 1:**
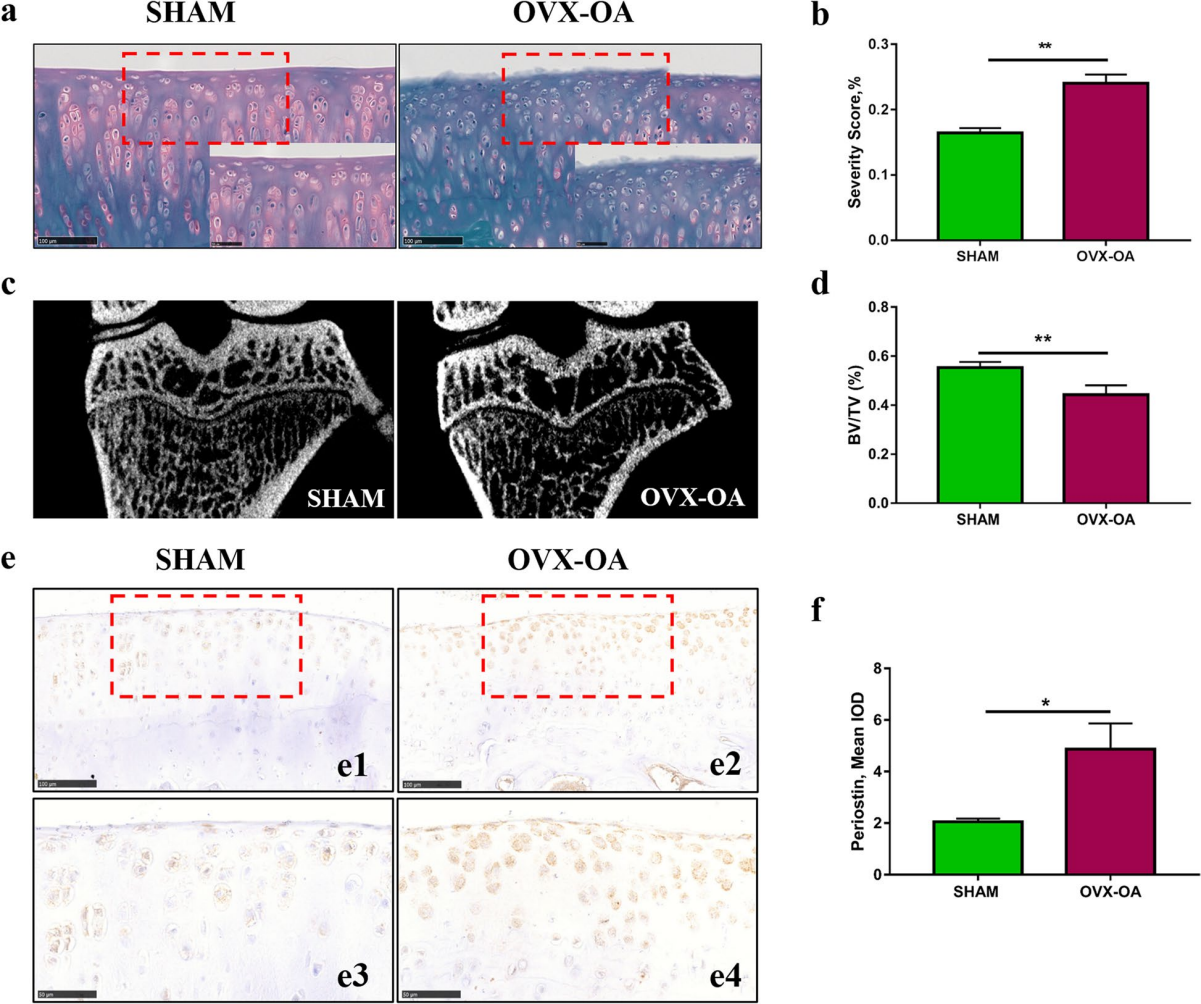
Evaluation of the OVX-OA model and periostin expression in the OVX-OA cartilage. **a** Representative safranin O-fast green stain images of the cartilage in Sham and OVX rats. **b** The erosion severity scores of cartilage in Sham and OVX rats. **c** Representative images of the subchondral bone in Sham and OVX-OA rats. **d** Quantitative analysis results of BV/TV of the subchondral bone in Sham and OVX-OA rats. **e** The representative periostin immunostaining images of the OVX-OA cartilage. Right panels (e3, e4) are high magnification images of the red frame in e1 e2, respectively. **f** The semi-quantitative result of periostin expression in the OVX-OA cartilage. OA, osteoarthritis; OVX, ovariectomy; BV/TV, bone volume/ total volume; IOD, integrated optical density

The OVX-OA model was used to histologically confirm the differential localization of POSTN in OA tissues. Based on Safranin O-Fast Green stain, the OVX rats shown significant proteoglycan loss and crude cartilage surface, while the relatively smooth cartilage surfaces was observed in SHAM rats (Fig. 1a). The OVX rats had significantly higher severity score than the SHAM rats (Fig. 1b). Furthermore, the trabecular parameters of subchondral bone were analyzed by micro-CT. The results showed that BV/TV was significantly lower in OVX-OA rats than in Sham rats (Fig. 1c-d), suggesting that osteoporosis was formed in OVX-OA rats and the OVX-OA model was successful.

The POSTN expression increased remarkably in the OVX rats compared to the Sham rats (Fig. 1e-f), which suggests that the POSTN might have been involved in the OA process.

### POSTN expression in the different compartments of OA clinical samples

**Fig. 2:**
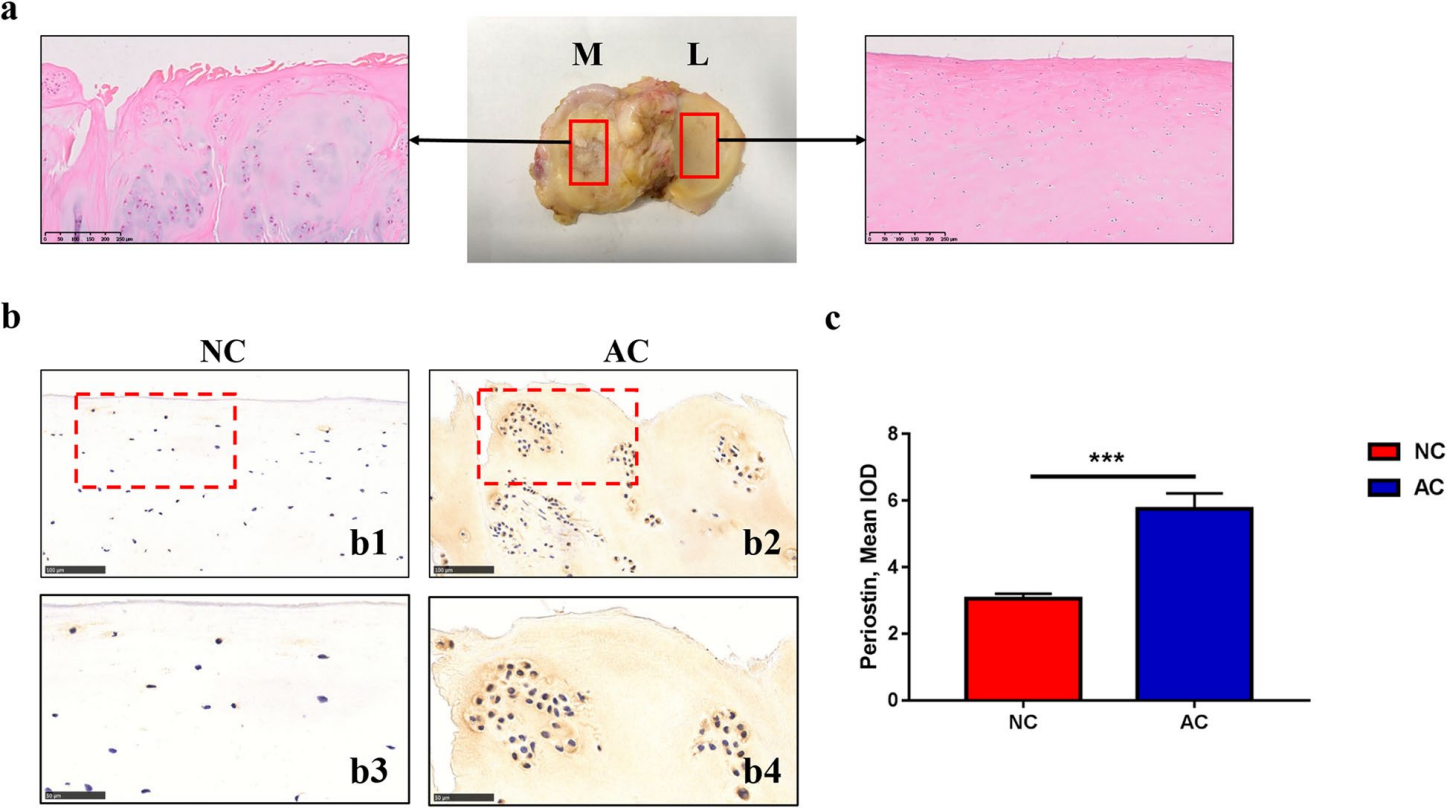
Periostin expression in human OA cartilage in different compartments. **a** Histological characterization of the clinical samples. Images in the black frame are the representative HE images of the areas in the red frame, bars = 250 µm. **b** Representative periostin immunostaining images of human OA cartilage. Right panels (b3 b4) are high magnification images of the red frames in b1 b2, respectively. The bars represent 100 µm in b1 b2 and 50 µm in b3 b4, respectively. **c** Semi-quantitative results of periostin expression in human cartilages. IOD, integrated optical density; M, medial compartment; L, lateral compartment; OA, osteoarthritis

As shown in Fig. 2, the positive staining of POSTN was scarcely detected in the intact area of the cartilage in the lateral plateau. However, more positive staining of POSTN was found in damaged cartilage in the medial plateau. The semi-quantitative results showed that the experimental group had significantly elevated expressions than the control group. These findings indicate the possibility that POSTN may not only take part in the onset and process of OA, but also in severity-related disease progression.

### Serum levels of POSTN in patients with and without OA

**Fig. 3:**
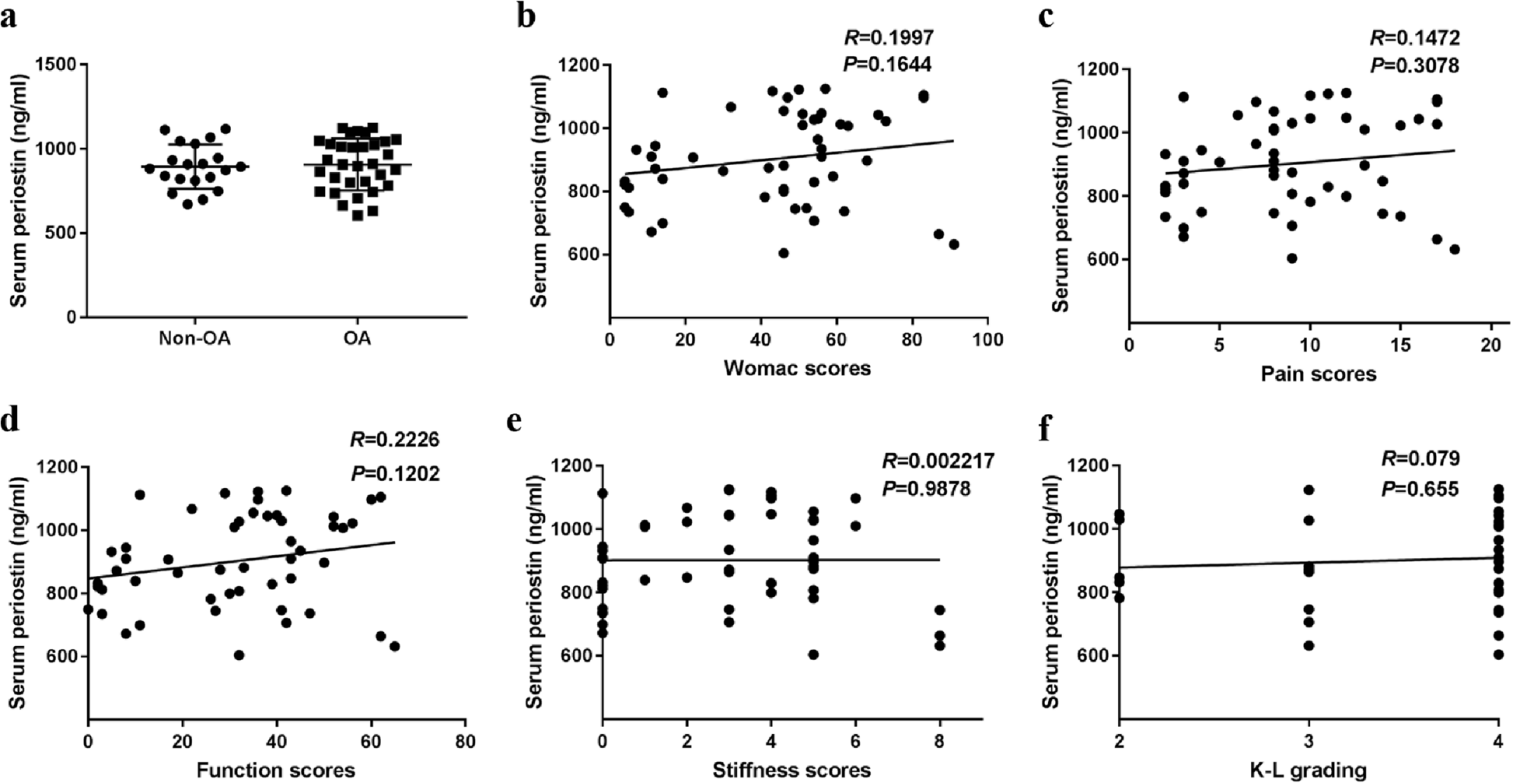
Correlations between serum periostin and osteoarthritic symptoms and radiographic severity. **a** Serum levels of periostin in patients with and without OA. Value = mean ± standard error of the mean. **b** Correlations between serum periostin and total Western Ontario and McMaster Universities Osteoarthritis Index scores. **c** Correlations between serum periostin and pain scores. **d** Correlations between serum periostin and function scores. **e** Correlations between serum periostin and stiffness scores. **f** Correlations between serum periostin and radiographic severity in all patients. OA, osteoarthritis

As shown in Fig. 3a, there was a slight increase in the serum POSTN of patients with OA compared to the controls. However, no significant differences were observed (894.2 ± 131.1 ng/mL vs 907.4 ± 153.1 ng/mL, *p* > 0.05) between patients with and without OA.

### Association between the serum POSTN and WOMAC scores

OA symptoms were evaluated using the WOMAC scores. No significant association was found between the serum POSTN levels and total WOMAC scores (*r* = 0.1997, *p* = 0.1644) (Fig. 3b-e). None of the WOMAC grading system subgroups (pain [*r* = 0.1472, *p* = 0.3078], stiffness [*r* = 0.002217, *p* = 0.9878], and function [*r* = 0.2226, *p* = 0.1202]) showed significant association with serum POSTN levels.

### Association between serum POSTN and Kellgren and Lawrence grade system

The KL severity score was used to assess the association between serum POSTN and radiological changes. Based on the radiographic KL classification, patients with OA were categorized into three subgroups as per OA grading (KL grade 2:5; KL grade 3:8; KL grade 4:22). No remarkable association was found between the serum POSTN level and K-L grading (*r* = 0.079, *p* = 0.655) (Fig. 3f).

### Risk factors as predictors of OA

**Fig. 4:**
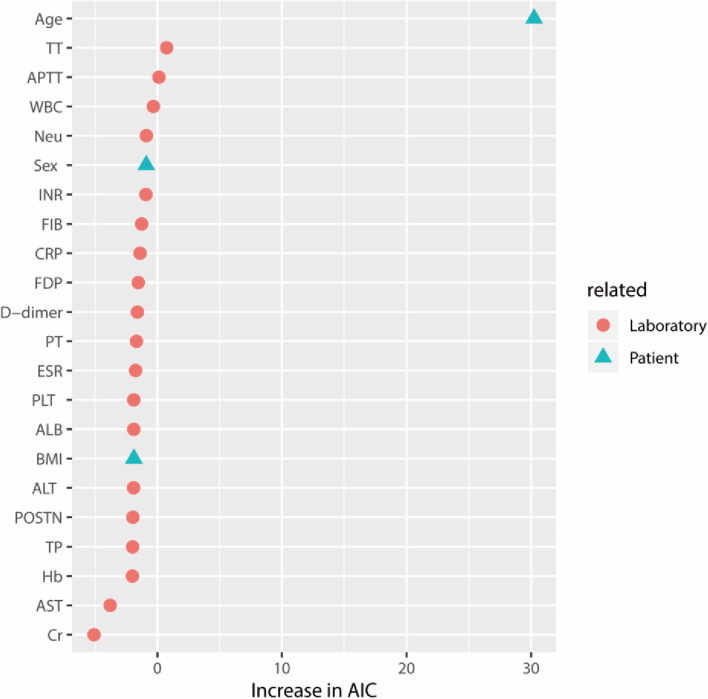
Relative variable importance of each predictor in the full model of OA grouped by risk factors related to laboratory and patient

We identified the predictive value of serum POSTN and several other risk factors that were potentially associated with OA. (Table 1) In model 1, the patient's age appeared to be a major risk predictor of knee OA (*p* < 0.001). When removing patient-related factors, serum POSTN seems was not a better laboratory-related factor in predicting knee OA (*p* > 0.05) (Table 1, Fig. 4). Additionally, when adding laboratory-related risk factors to patient-related risk factors, the AIC suggested that adding these covariates would not yield a remarkable improvement in predicting OA (the AIC did not change remarkably). Similarly, other possible patient-related risk factors did not increase the risk of OA.

## Discussion

POSTN was originally identified in osteoblasts and was found to play a role in mediating the transforming growth factor-β (TGF-β) signal pathway which is an important pathway to regulate cartilage metabolism. Therefore, POSTN is also actively involved in the pathogenesis of OA [28]. Although POSTN has been suggested to be involved in the pathogenesis of OA, few clinical studies have examined its potential value as a biomarker in diagnosing, distinguishing the severity, and predicting the risk of developing the disease. To the best of our knowledge, this is the first epidemiological study to investigate the value of POSTN as an OA biomarker from above all aspects.

First, we established the OVX-OA model to verify whether POSTN is involved in the OVX-OA process. The OVX model has been confirmed to induce OA [29–32], and the tear in OVX-OA cartilage develops slowly, with characteristics similar to the process in the early stages of human OA. In this study, we found increased POSTN expression in the articular cartilage of OVX-OA rats compared to Sham rats. This finding is consistent with previous studies showing the detrimental role of POSTN in promoting inflammation and cartilage catabolism [15, 33]. On the other hand, the POSTN deficiency has been suggested by Mukundan Attur et al. to protect against post-traumatic and age-related spontaneous OA [34]. These findings demonstrate that POSTN is involved in OVX-OA process.

Next, to confirm that POSTN was related to disease severity in the progression of OA, clinical samples from patients with OA were used. In the clinic, it can be found that a considerable portion of patients with OA has a predominantly torn cartilage in the medial compartment with intact cartilage in the lateral compartment [35]. The severely damaged cartilage in the medial compartment may reflect the late stage of the OA, and the less damaged cartilage in the lateral compartment may reflect the early stage of OA. The naturally different degrees of degradation in different compartments provided a good method to explore the different progression of the disease, eliminating the heterogeneity of different patients. In this study, the level of POSTN was highly expressed in the damaged cartilage of the medial compartment compared to the intact cartilage of the lateral compartment in clinical OA samples. This is in line with a previous study that reported that POSTN is mainly expressed near the erosive area of the cartilage in human OA [17]. The different expression of POSTN in different compartments may be involved in the disease severity of OA process.

The locally differential expression of POSTN suggested its participation in the progression of the disease and that it was valuable in disease diagnosis and distinguishing disease severity. However, the application value of local POSTN as a clinical biomarker of OA is limited. Because it is difficult and uneconomical to clinically harvest cartilage, invasive operations, which are necessary to harvest the local cartilage, can cause additional trauma and influence the operation time selection and postoperative infection of TKA that may be subsequently required [36]. On the contrary, the serum has better potential application value and is most commonly used in clinical settings.

Therefore, we further investigated whether the systemic level of serum POSTN could contribute to the diagnosis of OA. However, the data showed that the serum level of POSTN was not significantly different between patients with and without OA. The discrepancy between knee joint cartilage at the local level and serum level may be explained by the following reasons. First, the POSTN may undergo a clearance process from the local articular tissues to the serum, leading to an underestimation of its serum concentration [21]. Second, it has been reported that the tertiary structure of the FAS-1 domains could be modified by binding of a ligand [37], which may lead to a decrease in epitope recognition by antibodies in the POSTN assay. Moreover, the POSTN immunoassay could detect all isoforms of POSTN despite the presence of different isoforms, leading to a relatively higher level of POSTN, which was detected in local cartilage tissue.

To further determine whether the serum POSTN is in favor of distinguishing the severity of OA, we explored the association between POSTN and clinical characteristics of OA. The WOMAC score is the most commonly used disease-specific measurement of clinical symptoms in OA, and has 24 items with a total score and three subscales: pain, stiffness and physical function [38]. Our study demonstrated that serum POSTN was not associated with the total WOMAC score, pain and physical dysfunction. Several studies have shown a poor correlation between symptoms and radiographs in OA [39]. Thus, we further explored the relationship between serum POSTN and radiographs using the K-L grade system to evaluate anteroposterior weight-bearing knee radiographs of patients. We found no significant positive correlation between serum POSTN and K-L grade scores, suggesting that serum POSTN failed to reflect the severity of both symptoms and radiography in OA.

We performed multivariable cumulative link models to verify the predictive value of POSTN as a risk factor. Our results showed that except for age as a valuable risk factor for OA, all other investigated risk factors including serum POSTN did not have remarkable value for predicting OA. This finding is consistent with the clinical settings that up to date there was no valuable and applicable laboratory factor to accurately predict OA development [40].

Notably, there are some limitations in the study. First, the sample size was relatively limited (*n* = 50), which maybe be one of the possible reasons for the non-significantly statistical difference in the serum POSTN level. Therefore, studies involving in larger sample sizes are needed to verify our preliminary findings. Second, the participants included in the OA group of this study were in the advanced stage of the disease. Thus, the predictive value of serum POSTN in the early stage of human OA was unknown. Future longitudinal studies, especially in the early stage of OA, are required to confirm these findings. Moreover, the serum POSTN level in OVX induced OA or other OA models remains unknown, it would be desirable to investigate the systemic level of this factor in OA animal models.

In conclusion, we investigated the potential value of POSTN as an OA biomarker at the local and the serum level, in a rat animal model and clinical patients, and in all aspects of disease diagnosis, distinguishing the severity of the disease, and predicting the risk of developing the disease. Our results suggest that POSTN may be involved in the progression of OA, and local POSTN can be valuable for disease diagnosis and distinguishing the severity of the disease. However, its serum level is not sufficient to serve as a candidate biomarker of OA given the current technology for analysis.

## Supplementary Information

**Additional file 1: Supplementary Table 1. **The details of patients included in the serum analysis. BMI, body mass index; CRP, C-reactive protein; WOMAC, Western Ontario and McMaster Universities Osteoarthritis Index scores; KL, Kellgren-Lawrence (KL) grading scores. 

## Data Availability

The datasets used and/or analyzed during the current study are available from the corresponding author on reasonable request.

## Abbreviations

OA: Osteoarthritis
POSTN: Periostin
OVX: Ovariectomy
ELISA: Enzyme-linked immunosorbent assay
SD: Sprague–Dawley
ROI: Region of interest
TKA: Total keen joint arthroplasty
ASS: Arthroscopy surgery
WOMAC: Western Ontario and McMaster Universities Osteoarthritis Index
KL: Kellgren-Lawrence
AIC: Akaike information criterion
OR: Odds ratios
