# Supplementary material for: Serum periostin level is not sufficient to serve as a clinically applicable biomarker of osteoarthritis
Source: BMC Musculoskelet Disord. 2022 Dec 1;23:1039. doi: 10.1186/s12891-022-06017-x (PMC9714069; doi:10.1186/s12891-022-06017-x)
Supplement: Supplementary file 1 — Additional file 1: Supplementary Table 1. The details of patients included in the serum analysis. BMI, body mass index; CRP, C-reactive protein; WOMAC, Western Ontario and McMaster Universities Osteoarthritis Index scores; KL, Kellgren-Lawrence (KL) grading scores. [file 12891_2022_6017_MOESM1_ESM.docx]

Supplementary Table 1. The details of patients included in the serum analysis.

| **Number of**  **samples** | | **Age***  **(Year)** | **BMI**  **(Kg/m2)** | **CRP*******  **(mg/L)** | **Time since diagnosis***  **(month)** | **WOMAC**  **score *** | | **K-L grades*** |
| --- | --- | --- | --- | --- | --- | --- | --- | --- |
| **Non-OA** | **18** | **38.28 ± 3.55** | **26.46 ± 0.90** | **2.52 ± 0.95** | **13.10 ± 3.57** | **17.78 ± 3.86** | **0.94 ± 0.19** | |
| **OA** | **32** | **68.16 ± 1.08** | **27.28 ± 0.68** | **4.51 ± 1.15** | **103.70 ± 11.89** | **56.91 ± 2.55** | **3.59 ± 0.12** | |
